# Effect of High-Dose Zinc and Ascorbic Acid Supplementation vs Usual Care on Symptom Length and Reduction Among Ambulatory Patients With SARS-CoV-2 Infection: The COVID A to Z Randomized Clinical Trial

**DOI:** 10.1001/jamanetworkopen.2021.0369

**Published:** 2021-02-12

**Authors:** Suma Thomas, Divyang Patel, Barbara Bittel, Kathy Wolski, Qiuqing Wang, Anirudh Kumar, Zachary J. Il’Giovine, Reena Mehra, Carla McWilliams, Steve E. Nissen, Milind Y. Desai

**Affiliations:** 1 Heart and Vascular Institute, Cleveland Clinic, Cleveland, Ohio; 2 Neurologic Institute, Respiratory Institute, Lerner Research Institute, Cleveland Clinic, Cleveland, Ohio; 3 Department of Infectious Diseases, Cleveland Clinic Florida, Weston

## Abstract

**Importance:**

There is limited evidence regarding early treatment of novel severe acute respiratory syndrome coronavirus 2 (SARS-CoV-2) infection to mitigate symptom progression.

**Objective:**

To examine whether high-dose zinc and/or high-dose ascorbic acid reduce the severity or duration of symptoms compared with usual care among ambulatory patients with SARS-CoV-2 infection.

**Design, Setting, and Participants:**

This multicenter, single health system randomized clinical factorial open-label trial enrolled 214 adult patients with a diagnosis of SARS-CoV-2 infection confirmed with a polymerase chain reaction assay who received outpatient care in sites in Ohio and Florida. The trial was conducted from April 27, 2020, to October 14, 2020.

**Intervention:**

Patients were randomized in a 1:1:1:1 allocation ratio to receive either 10 days of zinc gluconate (50 mg), ascorbic acid (8000 mg), both agents, or standard of care.

**Outcomes:**

The primary end point was the number of days required to reach a 50% reduction in symptoms, including severity of fever, cough, shortness of breath, and fatigue (rated on a 4-point scale for each symptom). Secondary end points included days required to reach a total symptom severity score of 0, cumulative severity score at day 5, hospitalizations, deaths, adjunctive prescribed medications, and adverse effects of the study supplements.

**Results:**

A total of 214 patients were randomized, with a mean (SD) age of 45.2 (14.6) years and 132 (61.7%) women. The study was stopped for a low conditional power for benefit with no significant difference among the 4 groups for the primary end point. Patients who received usual care without supplementation achieved a 50% reduction in symptoms at a mean (SD) of 6.7 (4.4) days compared with 5.5 (3.7) days for the ascorbic acid group, 5.9 (4.9) days for the zinc gluconate group, and 5.5 (3.4) days for the group receiving both (overall *P* = .45). There was no significant difference in secondary outcomes among the treatment groups.

**Conclusions and Relevance:**

In this randomized clinical trial of ambulatory patients diagnosed with SARS-CoV-2 infection, treatment with high-dose zinc gluconate, ascorbic acid, or a combination of the 2 supplements did not significantly decrease the duration of symptoms compared with standard of care.

**Trial Registration:**

ClinicalTrials.gov Identifier: NCT04342728

## Key Points

**Question:**

Do high-dose zinc, high-dose ascorbic acid, and/or a combination of the 2 reduce the duration of symptoms of severe acute respiratory syndrome coronavirus 2 (SARS-CoV-2)?

**Findings:**

In this randomized clinical trial of 214 patients with confirmed SARS-CoV-2 infection receiving outpatient care, there was no significant difference in the duration of symptoms among the 4 groups.

**Meaning:**

These findings suggest that treatment with zinc, ascorbic acid, or both does not affect SARS-CoV-2 symptoms.

## Teaser

This randomized clinical trial examines whether high-dose zinc and/or high-dose ascorbic acid reduces the severity or duration of symptoms compared with usual care among ambulatory patients with severe acute respiratory syndrome coronavirus 2 (SARS-CoV-2) infection.

## Introduction

Severe acute respiratory syndrome coronavirus 2 (SARS-CoV-2) is a novel strain of enveloped RNA virus that has emerged as a deadly virus resulting in an international pandemic. Common symptoms at the onset of disease mimic influenza and include fever, nonproductive cough, myalgia, and fatigue.^1^ The US Centers for Disease Control and Prevention (CDC) list of SARS-CoV-2 symptoms includes fever or chills, cough, shortness of breath or difficulty breathing, fatigue, muscle or body aches, headache, new loss of taste or smell, sore throat, congestion or runny nose, nausea or vomiting, and diarrhea.^2^ In China, most patients (81%) with a confirmed diagnosis experienced only mild symptoms and did not require hospitalization or further care beyond supportive treatment.^3^ However, given the latency of the disease and prolonged incubation period, even patients who present with mild symptoms may progress to requiring hospitalization, prescription medication therapy, and mechanical ventilation and/or death.

Zinc gluconate and ascorbic acid are commonly available over-the-counter supplements that patients take for the treatment of viral illnesses. Zinc has been purported to increase polymorphonuclear cells’ ability to fight infection, and ascorbic acid is an antioxidant that may play a role in immune response.^4,5^ Limited evidence suggests that high doses of ascorbic acid and zinc gluconate may reduce duration of common cold symptoms and decrease the severity of symptoms.^6,7,8,9^ However, the role of zinc gluconate and ascorbic acid in decreasing symptoms and improving recovery in patients diagnosed with SARS-CoV-2 infection is uncertain. The current study sought to determine whether zinc and/or ascorbic acid reduces the severity or duration of symptoms associated with SARS-CoV-2 compared with usual care.

## Methods

The COVID A to Z study was a prospective randomized clinical open-label trial at multiple hospitals within a single health system, involving sites in Ohio and Florida. This study was approved by the Cleveland Clinic institutional review board and followed the Consolidated Standards of Reporting Trials (CONSORT) reporting guideline. All patients participating in the study provided written informed consent. Enrolled participants remained in their own home settings, and all study visits and/or procedures were conducted virtually, via telephone, email, computer, or laptop. The trial was designed to enroll approximately 520 adult patients diagnosed with SARS-CoV-2 infection with a polymerase chain reaction–based assay as outpatients who would likely remain in the outpatient setting for treatment. The full protocol of the study is available in Supplement 1.

**Figure 1.**
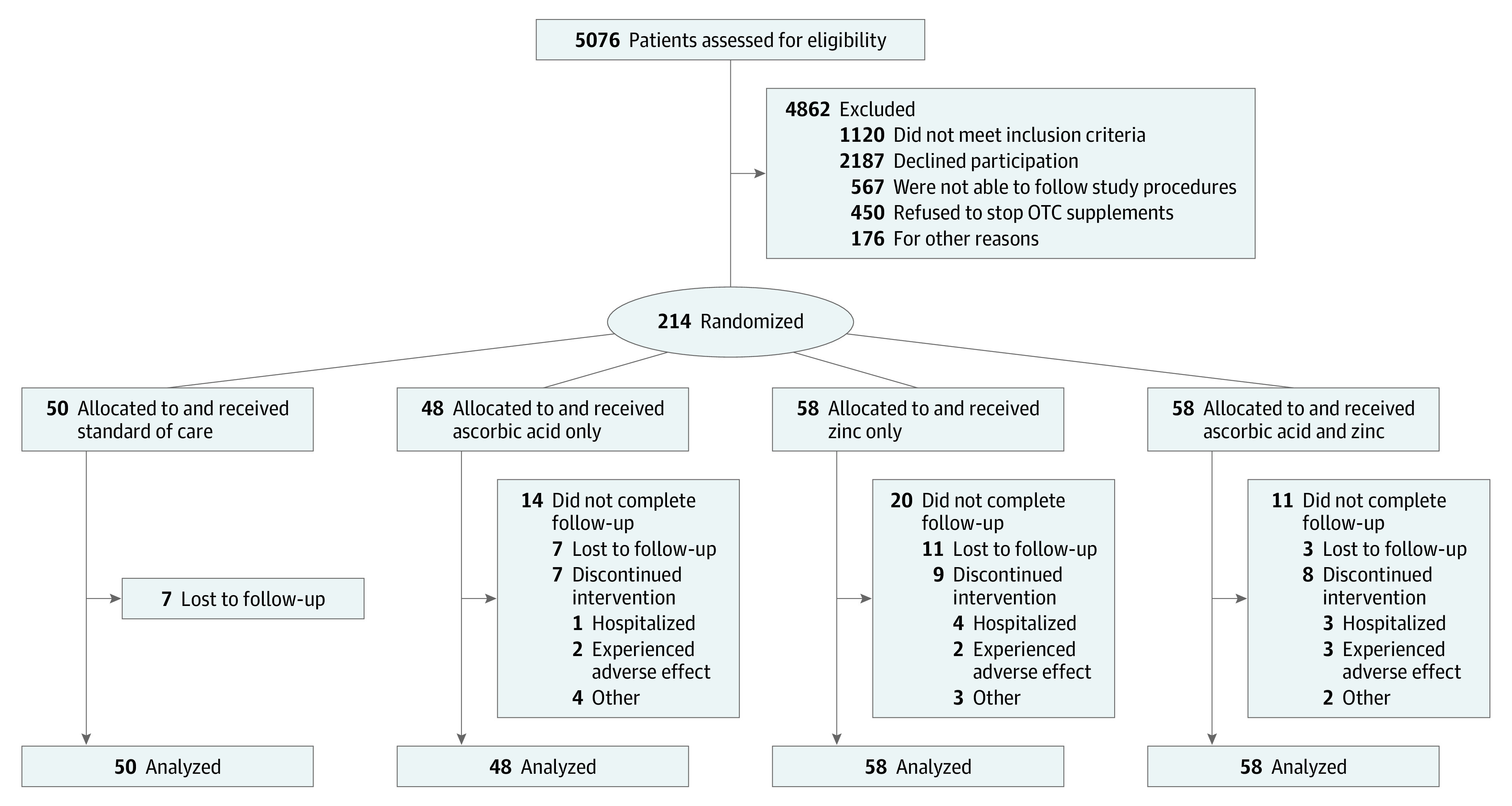
Patient Flow Diagram OTC indicates over the counter.

Patients were included in the study if they had a new diagnosis in an outpatient setting and were aged 18 years or older. Women of childbearing potential had to confirm a menstrual period within the past 30 days or previous sterilization, and those who were perimenopausal required a negative pregnancy test. Women of childbearing potential were required to have a confirmed negative pregnancy test to be enrolled. Patients were excluded if they were hospitalized, resided outside of Ohio or Florida, were pregnant, were actively lactating, or had advanced chronic kidney disease, liver disease awaiting transplantation, or a history of calcium oxalate kidney stones. Race/ethnicity were self-reported. The patient flow diagram is shown in Figure 1.

### Treatment Design

Patients were randomized in a 1:1:1:1 allocation strategy to 1 of 4 treatment strategies with a treatment duration of 10 days after a positive diagnosis. The 4 treatment strategies were as follows: (1) 8000 mg of ascorbic acid (to be divided over 2-3 times per day with meals), (2) 50 mg of zinc gluconate at bedtime, (3) both therapies, or (4) usual care without any study medications. The randomization grid was designed via the REDCap database and based on 25% of anticipated enrolled patients in each of the 4 groups. An automatically created link in REDCap randomized the patient to the supplement group based on the randomization grid.

Patients were asked to track their systemic illness daily based on symptoms. Patients were also asked to complete a questionnaire at the beginning of the study and every week after until day 28 to assess whether they were hospitalized or experienced adverse effects from the supplements. For each symptom, the patients assigned a score of 0 to 3 (with 0 indicating no symptoms; 1, mild symptoms; 2, moderate symptoms; and 3, severe symptoms). In the original analysis plan, patients recorded a questionnaire with only 4 symptoms (ie, fever/chills, shortness of breath, cough, and fatigue) for scores ranging from 0 to 12. However, based on CDC guidelines, the study protocol was amended on July 16, 2020, and the symptom questionnaire was expanded to include a total of 12 symptoms (ie, fevers/chills, shortness of breath, cough, fatigue, muscle or body aches, headache, new loss of taste, new loss of smell, congestion or runny nose, nausea, vomiting, and diarrhea), creating a score ranging from 0 to 36.^2^ The original 4-symptom scale was collected from all patients. The 12-symptom scale was only collected from patients enrolled after the July 16, 2020, amendment.

Patients were contacted by study coordinators weekly via email or daily via telephone to assess scores and hospitalizations, adverse effects, and additional medications. Patients seen in the emergency department (without an inpatient hospitalization) during the course of the study were asked to take study supplement(s) if the emergency department visit occurred within the initial 10 days of the study and to continue daily symptom assessments. Patients admitted to the hospital during the course of study were considered treatment failures and were no longer be required to continue study supplementation or track their daily symptoms.

### Trial End Points

The primary end point was the number of days required to reach a 50% reduction in symptom severity score from peak symptom score. This end point is reported for both the 4-symptom score available for all patients and the subset of patients for whom the 12-symptom score was available. Additional end points were the number of days required to reach a total symptom severity score of 0, cumulative severity score at day 5, hospitalizations, deaths, adjunctive prescribed medications, and adverse effects of the study supplements.

### Interim Analysis

An operational and safety monitoring board (OSMB) within the Cleveland Clinic was established in April 2020 to provide safety monitoring and evaluate operational performance of all SARS-COV-2–related studies at the Cleveland Clinic. None of the OSMB members were involved in the conduct of the study. Due to slower than expected enrollment, an interim analysis was conducted at approximately 40% of expected enrollment (214 of 520 patients). Stopping for superiority would only be considered if any treatment group achieved *P* < .001 compared with placebo. Stopping for futility would be considered if the conditional power was less than 30% for any (or all) treatment groups compared with placebo.

### Statistical Analysis

We assumed that the usual care group would achieve a 50% reduction in symptom severity in a mean (SD) of 6 (3) days and that at least 1 of the other 3 study groups would achieve a 50% reduction in a mean (SD) of 5 (3) days. Assuming a sample size in each of the 4 groups of 130 patients, a 1-way analysis of variance would have 80% power (2-sided α of .05) to detect a difference in means of 1 day with a common SD of 3 days.

Patients were categorized as either meeting the primary end point or failing to meet the primary end point. Patients who died or were hospitalized during the study were counted as treatment failures. The primary end point was defined as the number of days from the time of peak symptom score to a 50% resolution in those achieving a 50% reduction within the study time frame. Patients who were asymptomatic at baseline were classified as missing when calculating the days to a 50% reduction in symptom score. In a sensitivity analysis, the number of days to reach a 50% reduction was set to 28 days for patients considered treatment failures. The original analysis plan was to evaluate all pairwise treatment comparisons with adjustment for multiple comparisons using the Tukey method. Because the study was stopped early for futility, the overall F-test *P* value from an analysis of variance is reported for all end points, summarizing number of days to 50% reduction. Nominal *P* values from the χ^2^ statistic are reported for categorical variables. Kaplan-Meier curves were created comparing the primary end point among the 4 treatments. The Kaplan Meier plot and *P* value from the log-rank test of the null hypothesis of no difference between the 4 survival curves were made using the survminer package in R version 3.6.1 (R Project for Statistical Computing). Statistical significance was set at *P* < .05, and all tests were 2-tailed.

## Results

### Early Stopping

The OSMB met on October 23, 2020, and recommended stopping the study for futility. The futility criteria was met for the 3 active treatment groups compared with the usual care group. Data on the 214 patients enrolled at the time of study termination are the final data for this study.

### Baseline Characteristics

**Figure 2.**
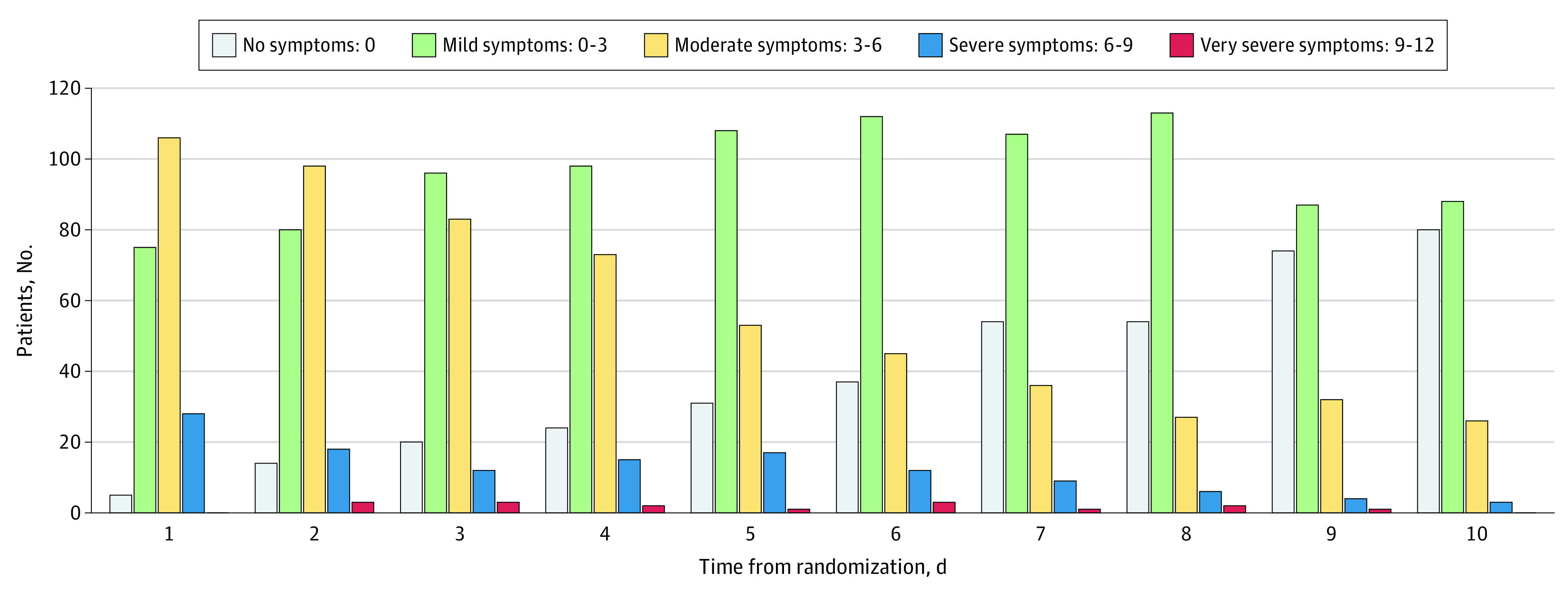
Patients Experiencing Symptoms by 4-Symptom Scale

A total of 214 patients were enrolled and randomized from April 27, 2020, to October 14, 2020. Of the 214 patients, 50 (23.4%) were randomized to usual care, 48 (22.4%) were randomized to ascorbic acid only, 58 (27.1%) were randomized to zinc gluconate only, and 58 (27.1%) to both supplements. Baseline characteristics of study participants are reported in Table 1. The mean (SD) age of study participants was 45.2 (14.6) years. There were 132 (61.7%) women in the study and 68 people (31.8%) reported currently or formerly smoking. At least one-quarter of participants used vitamins and minerals previously (56 [26.2%]). The mean (SD) symptom composite score (of 12 possible points) at baseline was 4.3 (1.9) points and was similar across treatment groups (Figure 2). In the subset of patients with a 12-symptom score (36 possible points) the mean (SD) was 11.6 (5.6) points.

**Table 1.** Baseline Characteristics of the Enrolled Population

Characteristic	No. (%)				
	Total (N = 214)	Standard of care (n = 50)	Ascorbic acid only (n = 48)	Zinc only (n = 58)	Ascorbic acid with zinc (n = 58)
Age, mean (SD), y	45.2 (14.6)	42.0 (14.6)	45.6 (15.0)	44.1 (14.8)	48.7 (14.3)
Women	132 (61.7)	31 (62.0)	33 (68.8)	37 (63.8)	31 (53.4)
Race					
White	152 (71.7)	31 (62.0)	39 (81.3)	42 (72.7)	40 (69.0)
Black	51 (23.8)	16 (32.0)	7 (14.6)	12 (20.7)	16 (27.6)
Other	2 (0.9)	0	1 (2.1)	0	1 (1.7)
Not reported	9 (4.2)	3 (6.0)	1 (2.1)	4 (6.9)	1 (1.7)
Body mass index, median (IQR)^a^	30.0 (26.2-36.6)	30.9 (25.8-38.2)	28.3 (25.8-32.0)	28.8 (26.2-37.9)	31.0 (27.7-36.5)
History					
Diabetes	29 (13.6)	3 (6.0)	2 (4.2)	7 (12.1)	17 (29.3)
Hypertension	70 (32.7)	14 (28.0)	8 (16.7)	21 (36.2)	27 (46.6)
Dyslipidemia	56 (26.2)	9 (18.0)	11 (22.9)	12 (20.7)	24 (41.4)
Asthma	33 (15.4)	7 (14.0)	8 (16.7)	10 (17.2)	8 (13.8)
Anxiety	39 (18.2)	11 (22.0)	13 (27.1)	8 (13.8)	7 (12.1)
Depression	33 (15.4)	8 (16.0)	7 (14.6)	10 (17.2)	8 (13.8)
Current or former smoking	68 (31.8)	14 (28.0)	17 (35.4)	16 (27.6)	21 (36.2)
Concomitant medications					
Antipyretics	59 (27.6)	17 (34.0)	9 (18.8)	16 (27.6)	17 (29.3)
NSAIDs	33 (15.4)	8 (16.0)	4 (8.3)	9 (15.5)	12 (20.7)
Bronchodilator	31 (14.5)	10 (20.0)	4 (8.3)	10 (17.2)	7 (12.1)
Gastrointestinal medications	22 (10.3)	4 (8.0)	6 (12.5)	7 (12.1)	5 (8.6)
Corticosteroids	18 (8.4)	4 (8.0)	5 (10.4)	6 (10.3)	3 (5.2)
Decongestant	14 (6.5)	3 (6.0)	3 (6.3)	5 (8.6)	3 (5.2)
Baseline composite COVID-19 symptom score, average points, median (IQR)					
4-component score^b^	4.0 (3.0-5.0)	4.0 (3.0-5.0)	4.0 (3.0-6.0)	4.0 (3.0-5.0)	4.0 (3.0-6.0)
12-component score^c^	11.0 (7.0-15.0)	11.0 (9.5-16.0)	12.5 (7.0-18.0)	8.0 (6.0-13.0)	11.0 (7.0-14.0)

Abbreviations: COVID-19, coronavirus disease 2019; IQR, interquartile range; NSAID, nonsteroidal anti-inflammatory drug.

^a^ Body mass index was calculated as weight in kilograms divided by height in meters squared.

^b^ The 4-component score was collected for all patients.

^c^ The 12-component score was collected for 78 patients who consented after July 16, 2020 (20 patients [25.6%] in standard of care, 14 patients [17.9%] in ascorbic acid only, 21 patients [26.9%] in zinc only, and 23 patients [29.5%] in the combined ascorbic acid and zinc group).

### Primary Outcome

**Figure 3.**
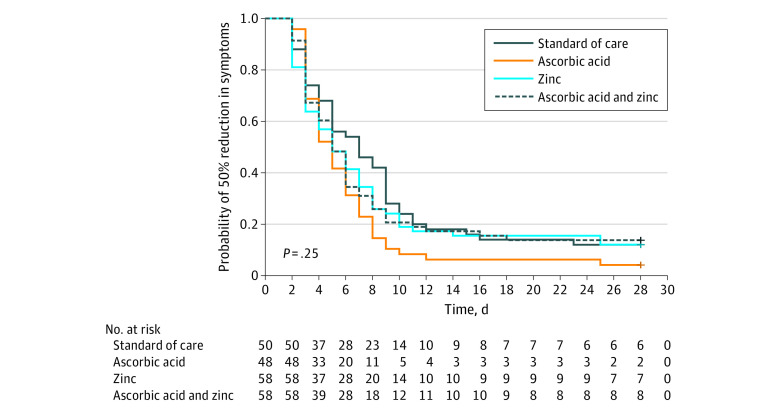
Kaplan-Meier Curves for the Primary End Point by Treatment Group

There was no significant difference in the primary outcome of days required to reach a 50% reduction in symptoms among the 4 study groups. Patients who received usual care without supplementation achieved a 50% reduction in symptoms in a mean (SD) of 6.7 (4.4) days compared with a mean (SD) of 5.5 (3.7) days for patients receiving ascorbic acid, a mean (SD) of 5.9 (4.9) days for patients receiving zinc gluconate, and a mean (SD) of 5.5 (3.4) days for patients receiving both ascorbic acid and zinc gluconate supplementation (overall *P* value = 0.45; log-rank *P* = .25) (Figure 3).

### Secondary Outcomes

There was no significant difference in any of the secondary outcomes, including number of days to reach no presence of fever, cough, shortness of breath, or fatigue. The mean (SD) composite 4-symptom score at day 5 was 3.2 (2.2) points and did not differ among the 4 study groups. A total of 17 patients (7.9%) were hospitalized before the 28-day study period ended, and 3 patients (1.4%) died after enrollment in the study (Table 2). However, both the number of hospitalizations and deaths did not significantly vary among the 4 treatment groups. Less than 3% of the population had medications added to treat coronavirus disease 2019 (COVID-19)–related symptoms, and less than 10% of the population experienced an adverse effect related to the supplement, with slightly more adverse effects, including nausea, diarrhea, and stomach cramps, in the group receiving ascorbic acid only (eTable in Supplement 2).

**Table 2.** Primary and Secondary End Points

End point	Mean (SD)					*P* value
	Total	Standard of care	Ascorbic acid only	Zinc only	Ascorbic acid with zinc	
**Primary end points**						
4-Symptom scale						
Patients meeting 50% reduction, No./total No. (%)	191/214 (89.3)	44/50 (88.0)	46/48 (95.8)	51/58 (87.9)	50/58 (86.2)	.41
Time to 50% reduction, d	5.9 (4.1)	6.7 (4.4)	5.5 (3.7)	5.9 (4.9)	5.5 (3.4)	.38^a^
Difference, d (95% CI)	NA	[Reference]	−1.18 (−2.88 to 0.51)	−0.86 (−2.76 to 1.03)	−1.3 (−2.86 to 0.32)	NA
12-Symptom scale						
Patients meeting 50% reduction, No./total No. (%)	74/75 (98.7)	18/19 (94.7)	14/14 (100.0)	21/21 (100.0)	21/21 (100.0)	.44
Time to 50% reduction, d	6.4 (3.3)	6.2 (2.9)	6.6 (3.7)	6.6 (3.7)	6.2 (3.2)	.97
Difference, d (95% CI)	NA	[Reference]	0.40 (−1.99 to 2.80)	0.40 (−1.77 to 2.58)	0.07 (−1.94 to 2.09)	NA
**Secondary end points**						
Time until 4-symptom composite score is 0						
Time, d	10.6 (6.1)	9.9 (4.4)	12.1 (6.9)	10.8 (6.8)	9.7 (5.7)	.29
Difference, d (95% CI)	NA	[Reference]	2.22 (−0.58 to 5.02)	0.85 (−1.91 to 3.62)	−0.24 (−2.67 to 2.19)	NA
Individual components, d						
No fever	2.3 (2.3)	2.2 (1.8)	2.5 (2.3)	1.9 (1.8)	2.7 (3.1)	.28
No cough	7.3 (6.2)	5.7 (4.9)	7.3 (6.6)	7.8 (6.6)	8.1 (6.5)	.30
No shortness of breath	3.9 (4.8)	3.6 (45)	4.5 (5.1)	3.4 (4.9)	4.2 (4.8)	.65
No fatigue	7.9 (6.0)	8.7 (5.5)	8.3 (6.4)	6.9 (6.1)	8.0 (6.0)	.56
Composite 4-symptom score at day 5						
Score	3.2 (2.2)	3.1 (2.3)	3.3 (2.1)	3.2 (2.2)	3.3 (2.3)	.94
Difference, d (95% CI)	NA	[Reference]	0.27 (−0.64 to 1.18)	0.11 (−0.78 to 1.00)	0.20 (−0.72 to 1.12)	NA
Hospitalization, No. (%)	17 (7.9)	3 (6.0)	2 (4.2)	5 (8.6)	7 (12.1)	.50^b^
Death, No. (%)	3 (1.4)	0	1 (2.1)	0	2 (3.4)	.40^b^

Abbreviation: NA, not applicable.

^a^ *P *value derived from nonparametric test.

^b^ *P *value derived from exact test.

### Serious Adverse Events

The data safety monitoring board noted 4 serious adverse events, including 3 patients who died from COVID-19 and another patient who was admitted to the hospital for a chronic obstructive pulmonary disease exacerbation during the study period. The board did not believe that any of the adverse events were caused by individual treatments that patients received as a part of the study.

## Discussion

The COVID A to Z study was designed to examine whether patients treated with zinc gluconate, ascorbic acid, or a combination of both treatments would experience a shortened duration of symptoms associated with SARS-CoV-2 compared with usual care. A significantly faster reduction in symptoms was not observed in any of the active treatment groups vs usual care. Based on an interim analysis, the study was stopped for futility.

The data about oral ascorbic acid and zinc are inconsistent, with some trials suggesting that high doses of ascorbic acid and zinc gluconate may reduce the duration of common cold symptoms and decrease the severity of symptoms, while other studies have shown no benefit.^6,7,8,9^ The data for intravenous ascorbic acid is also variable, with a meta-analysis review that investigated the role of ascorbic acid in critically ill patients^10^ showing no significant association with mortality but variable associations with secondary end points, including duration of ventilator support and hospital length of stay. Ongoing clinical trials in China and the United States are investigating the potential role of intravenous ascorbic acid in reducing respiratory failure requiring mechanical ventilation in patients with SARS-CoV-2. In addition, ascorbic acid, zinc, and vitamin D are being studied for the prevention of SARS-CoV-2 infection. What is unknown is whether ascorbic acid and zinc gluconate can shorten the duration or prevent progression of the disease.

In terms of biologic plausibility, zinc is known to play a role in immune function via antibody and white blood cell production.^4^ Zinc supplementation has been suggested to increase polymorphonuclear cells’ ability to fight infection, while there is evidence that zinc deficiency increases pro-inflammatory cytokines and decreases the production of antibodies. Zinc has also been implicated in coronavirus biology.^11^ Angiotensin-converting enzyme 2 is a zinc metalloprotease that is important for cellular entry of coronavirus.^12^ In addition, studies on SARS, a coronavirus, have shown that zinc can inhibit its ribonucleic acid polymerase.^11^ However, the biologic activity of zinc against viruses may require ionophores, such as pyrithione, to block viral replication.^13^ Ascorbic acid is known to be an antioxidant, and a variety of studies have suggested that it can affect the immune system.^14^ Moreover, in vitro and in vivo studies in avians have shown that ascorbic acid could be protective against coronavirus, and human trials have found that it may decrease susceptibility to viral respiratory infections and pneumonia.^15^

However, based on the current study, these supplements cannot be recommended to reduce symptom morbidity in such patients. High-dose zinc gluconate, ascorbic acid, or both supplements did not reduce SARS-CoV-2 symptoms. Most consumers of ascorbic acid and zinc are taking significantly lower doses of these supplements, so demonstrating that even high-dose ascorbic acid and zinc had no benefit suggests clear lack of efficacy. In addition, administering supplements with unproven benefit can be detrimental due to adverse effects. Zinc has been shown to cause a metallic taste, dry mouth, and gastrointestinal intolerance in high doses.^16^ Ascorbic acid can cause gastrointestinal intolerance, and in the current study, a significantly higher proportion of patients in the ascorbic acid subgroups reported adverse effects, including nausea, diarrhea, and stomach cramps.

### Strengths and Limitations

There are several strengths and limitations that should be acknowledged. A major strength is the pragmatic design of the study and its novel primary end point, which was based on a symptom assessment questionnaire (time to reduction in symptom score by 50%). A major limitation was that there was no placebo control group; the current study was open label, and patients were not masked to which therapy they received. Patients were recruited in a single health system, and therefore, the outcomes in our health system may not represent the outcomes of patients in other health care settings. However, it should be acknowledged that patients were recruited from multiple outpatient facilities in Ohio and Florida. It is possible that certain groups with higher susceptibility (eg, older patients and patients from minority racial/ethnic groups) were underrepresented in the current study and the results may not be broadly generalizable. Also, stratification of symptoms by age, sex, race, or duration of symptoms prior to testing were not taken into consideration in the current analysis. Furthermore, the doses of zinc and ascorbic acid, while well tolerated, could be lower than amounts needed to shorten the duration of symptoms, and patients could have previously taken supplements such as zinc and ascorbic acid before enrolling in the study. Recent studies have also demonstrated that vitamin D deficiency is associated with increased risk of SARS-CoV-2 infection and an increased risk of hospitalization,^17^ so the potential role of other supplements in decreasing SARS-CoV-2 symptoms cannot be concluded from our study. Randomized trials are currently being performed to answer whether vitamin D supplementation can benefit patients diagnosed with SARS-CoV-2.

## Conclusions

In this randomized clinical trial, ambulatory patients diagnosed with SARS-CoV-2, treatment were treated with high doses of zinc gluconate, ascorbic acid, or a combination of zinc gluconate and ascorbic acid. These interventions did not significantly shorten the duration of symptoms associated with the virus compared with usual care.

## Notes

Supplement 1. **Trial Protocol**

Supplement 2. **eTable. **Adverse Effects in the Study Population

Supplement 3. **Data Sharing Statement**
