# Supplementary material for: Effect of High-Dose Zinc and Ascorbic Acid Supplementation vs Usual Care on Symptom Length and Reduction Among Ambulatory Patients With SARS-CoV-2 Infection: The COVID A to Z Randomized Clinical Trial
Source: JAMA Netw Open. 2021 Feb 12;4(2):e210369. doi: 10.1001/jamanetworkopen.2021.0369 (PMC7881357; doi:10.1001/jamanetworkopen.2021.0369)
Supplement: Supplement 1. — Trial Protocol [file jamanetwopen-e210369-s001.pdf]

CORONAVIRUS DISEASE 2019- USING ASCORBIC ACID AND ZINC SUPPLEMENTATION (COVIDAtoZ) Research Study

A RANDOMIZED, OPEN LABEL SINGLE CENTER STUDY

Version Date: June 30, 2020

Investigators

Milind Desai, MD (Co-Principal Investigator)

Suma Thomas, MD (Co-Principal Investigator)

Divyang Patel, MD (Co-Investigator)

Barbara Bittel, BSN, RN (Co-Investigator)

Anirudh Kumar, MD (Co-Investigator)

Kathy Wolski, MPH (Co-investigator)

Steve E Nissen, MD (Co-Investigator)

Sponsor: Cleveland Clinic Heart, Vascular and Thoracic Institute

PROTOCOL SIGNATURE PAGE

I have carefully read the COVIDAtoZ Research Study protocol. I agree to conduct this study as outlined herein. Furthermore, I understand that the Cleveland Clinic and the IRB must approve any changes to the protocol in writing before implementation.

_______________________________ Date ______________________

Milind Desai, MD

_________________________________ Date ______________________

Suma Thomas, MD, MBA

54 **Study Synopsis**

Title	Coronavirus Disease 2019- Using Ascorbic acid and Zinc Supplementation (**CovidAtoZ**) Research Study
Purpose	The primary objective of this study is to determine if ascorbic acid (vitamin C) and zinc gluconate result in shortening the duration and type of symptoms and prevention of hospitalizations in patients with confirmed new diagnosis of coronavirus disease 2019 (COVID-19)
Design	A prospective, open label four arm study (1. Zinc only 2. Zinc and ascorbic acid 3. Ascorbic acid only 4. Standard of care. All subjects will receive the supplements for 10 days after positive test result
Number of Subjects	520
Inclusion Criteria	Outpatients ≥ 18 years presenting to Cleveland Clinic Health System in Ohio and Florida who test positive for COVID-19 having any of the following symptoms<br>1. Fever or chills<br>2. Shortness of breath or difficulty breathing<br>3. Cough<br>4. Fatigue<br>5. Muscle or body Aches<br>6. Headache<br>7. New loss of taste<br>8. New loss of smell<br>9. Congestion or runny nose<br>10. Nausea<br>11. Vomiting<br>12. Diarrhea<br>Women of child bearing potential:<br>1. have had a menstrual period within the past 30 days, or<br>2. have had previous sterilization, or<br>3. are perimenopausal (less than 1 year) who have a negative pregnancy test, or<br>4. women of childbearing potential who do not meet the above and have a negative pregnancy test.
Exclusion Criteria	Patients who are found to be positive during hospitalization<br>Patients who reside outside Ohio or Florida.<br>Pregnant women:

	Current known pregnancy<br>Positive pregnancy test (women of child bearing potential who have not had previous sterilization as defined as hysterectomy or tubal ligation)<br>Women of childbearing potential who do not meet the above criteria, last menstrual period greater than 30 days and have a positive pregnancy test.<br>Lactating Women<br>End stage kidney disease<br>Advanced liver disease awaiting transplant<br>History of Calcium Oxalate kidney stones.
Duration of Participation	Approximately 4 weeks after enrollment
Study Centers	Cleveland Clinic Main Campus Cleveland, Ohio and Florida including Satellite/ Regional Healthcare Facilities
Primary Endpoint	Duration of symptoms from coronavirus 2019 including fever or chills, shortness of breath or difficulty breathing, cough, fatigue, chills, muscle or body aches, headache, new loss of taste, new loss of smell, congestion or runny nose, nausea, vomiting and/or diarrhea.
Secondary Endpoints	Need for prescription medication, need for ER visits, need for hospitalization, side effects from supplementation

1.0 INTRODUCTION

1.1 Purpose of this Study:

The purpose of this study is to examine the impact of ascorbic acid (vitamin c) and zinc gluconate in reducing duration of symptoms in patients diagnosed with coronavirus disease 2019 (COVID-19). Patients above the age of 18 who present to the Cleveland Clinic (Ohio & Florida) emergency room or outpatient testing and receive a positive test for COVID-19 who are remaining in the outpatient setting for treatment, will be invited to participate in the study and included if they consent to participate. They will be randomized to receive either ascorbic acid, zinc gluconate, combination of ascorbic acid and zinc gluconate supplements or standard of care

(none of these additional supplements). These supplements will be given to patients for a duration of ten days as a part of the study. Patients will be asked to track their symptoms of systemic illness on a daily basis.(Appendix A: Symptom Assessment Tool). We will track 12 symptoms on a scale of 0-3 as follows: A) Temperature Scale (if patient has a thermometer): 0 = ≤98.6, 1 = >98.6- 100.6, 2 = > 100.6 – 102.6, 3 = >102.6; If no thermometer: 0= No fever & no chills, 1= No fever & chills, 2= Fever & no chills, 3= Fever & chills B) Cough Scale: 0 = no cough, 1 = mild, 2 = moderate, 3 = severe C) Shortness of breath: 0 = no shortness of breath, 1 = with moderate intensity exercise 2 = with walking on flat surface 3 = short of breath with getting dressed or daily activities D) Fatigue: 0 = No fatigue/energetic, 1=mild fatigue, 2=moderate fatigue, 3=severe fatigue E) Muscle or body aches: 0 = No muscle or body aches, 1=mild muscle or body aches, 2=moderate muscle or body aches, 3=severe muscle or body aches. F) Headache: 0 = No headache, 1=mild headache, 2=moderate headache, 3=severe headache G) New loss of taste: 0 = No loss of taste, 1=mild loss of taste, 2=moderate loss of taste, 3=severe loss of taste H) New loss of smell: 0 = No loss of smell, 1=mild loss of smell, 2=moderate loss of smell, 3=severe loss of smell I) Congestion or runny nose: 0 = No congestion or runny nose, 1=mild congestion or runny nose, 2=moderate congestion or runny nose, 3=severe congestion or runny nose J) Nausea: 0 = No nausea, 1=mild nausea, 2=moderate nausea, 3=severe nausea K) Vomiting: 0 = No vomiting, 1=mild vomiting, 2=moderate vomiting, 3=severe vomiting L) Diarrhea: 0 = No diarrhea, 1=mild diarrhea, 2=moderate diarrhea, 3=severe diarrhea. Each patient will have a composite score ranging from 0-12/day. The tracking will continue for the 28 days of study participation for each subject. They will also be asked to complete a questionnaire at the beginning of study period, day 7, day 14, day 21 and day 28. The purpose of the

questionnaire is to assess whether patient received additional medications/ supplements, sought treatment at emergency rooms or were hospitalized, or experienced side effects from the study supplements.

The primary clinical endpoint is the number of days required to reach a 50 percent reduction in the cumulative 0-36 score from the four symptom categories. For secondary endpoints we will assess a) the number of days required to reach a score of 0 b) total composite score at day 5 of the supplementation, c) differences in emergency room visits and d) differences in hospitalizations e) differences in severity of symptoms between study arms f) differences in number of patients who were prescribed adjunctive medications for their diagnosis, and g) differences in number of patients in study arms who experienced side effects from the supplements.

1.2 Importance and Rationale of Study

COVID-2019 is a novel strain of enveloped RNA virus that emerged as a deadly virus from Wuhan, China and led to an international pandemic. Common symptoms at the onset of disease mimic influenza and include fevers, non-productive cough, myalgia, and fatigue.[1] The largest experience to date comes from China where the majority of patients (81%) with a confirmed diagnosis of coronavirus 2019 experienced only mild symptoms and did not require hospitalization or further therapy beyond support treatment.[2] However given the latency of the disease and prolonged incubation period even patients who present with mild symptoms may progress to requiring hospitalization, prescription medication therapy, mechanical ventilation, and/or even death.

Currently, there are no FDA approved therapies for the symptom management of COVID-2019. Limited data suggests that hydroxychloroquine, remdesivir, tocilizumab, and convalescent antibody may have a role on the impact of symptoms in patients affected with the disease. However, besides hydroxychloroquine the other therapies are limited to patients with severe manifestations of the disease including development of acute respiratory distress syndrome and require approval from institutional review boards, institutions, companies, and/or the federal government to receive therapy. IV Ascorbic acid is being studied for the effects on symptoms in patients with COVID-2019 in China and the United States. Given the limited therapy available for patients, we wanted to study the impact of high dose of ascorbic acid and zinc gluconate on symptoms of COVID-2019 in patients who present in and receive a confirmed diagnosis.

Biological Effects/Feasibility

Zinc is known to be important for immune function and has a role in antibody and white blood cell production. Zinc supplementation has been shown to increase polymorphonuclear cells' ability to fight infection, while there is evidence that zinc deficiency increases pro-inflammatory cytokines and decreases the production of antibodies. Zinc has also been implicated in coronavirus biology. Angiotensin-Converting-Enzyme 2 is a zinc metalloprotease that is important for cellular entry of coronavirus. In addition, studies on SARS, a coronavirus, have shown that zinc can inhibit its RNA polymerase.

Vitamin C is known to be an antioxidant and a variety of studies have suggested that vitamin C can impact the immune system. Moreover, in vitro and in vivo studies in avians have shown that vitamin C could be protective against coronavirus and human trials have found that

vitamin C may decrease susceptibility to viral respiratory infections and pneumonia. As a result of these biologic effects, we hypothesize zinc and vitamin C supplementation may have a potential role in improving the response to an infection such as COVID-2019.

The purpose of the current study is to see whether ascorbic acid and zinc gluconate which has limited side effect profile and is readily available over the counter can decrease the duration of symptoms seen in patients with new diagnosis of COVID-2019. A secondary purpose is to see whether Zinc and/or Ascorbic acid supplementation can prevent progression of the severe manifestations of the disease including development of dyspnea and acute respiratory distress syndrome which may require hospitalization, mechanical ventilation, and or lead to death.

1.3 Brief summary of relevant literature:

A Medline literature search with the following unique search terms; coronavirus 2019 was performed.

The relevant literature was summarized in the rationale section above. Briefly, there is variable data about ascorbic acid and vitamin C, but there is evidence that high doses of ascorbic acid (vitamin C) and zinc gluconate reduce duration of common cold symptoms and decrease the severity of symptoms.[3-6] Furthermore, it is known that majority of patients infected with coronavirus 2019 have mild symptoms that do not require any therapy besides supportive care. In addition, a review of a meta-analysis investigating the role of IV vitamin C in critically ill patients showed no significant effect on mortality but showed variable effects on secondary endpoints including duration of ventilator support and hospital length of stay.[7] What is unknown is whether ascorbic acid and zinc gluconate can shorten the duration of the disease and whether it can prevent progression of the disease.

2.0 Study Design, Objective, and Clinical Endpoints

This is a prospective, randomized study which plans to enroll 520 patients with a principal diagnosis of COVID-2019, managed in an outpatient setting, who presented after being sent by a healthcare provider to get tested and receive a PCR-assay based confirmed diagnosis of the disease. All patients who agree to participate in the study will answer a baseline questionnaire about their symptoms at the time of inclusion. Patients will then be randomized to one of 4 study arms. Patients in Arm A (n=130) will receive 8000 mg of vitamin C (to be taken divided over 2-3 times a day with meals) for 10 days after positive diagnosis. Patients in Arm B (n=130) will receive zinc gluconate 50 mg for 10 days, to be taken at bedtime, after positive diagnosis. Patients in Arm C (n=130) will receive 8000 mg of vitamin C (to be taken divided over 2-3 times a day with meals) and zinc gluconate 50 mg for 10 days (taken at bedtime). Patients in Arm D (n=130) will not receive any of the study medications and continue on standard of care.

Patients will then track their symptoms daily from day 0 to day 28 answering 12 basic questions on illness severity. Symptom assessment tools will be completed by patients daily either electronically or on paper. For those completing the tool electronically a tool will be emailed to the patient daily via RedCap. For those completing the tool on paper a 28 day paper assessment tool will be provided to the patient at the time of consent via email/ courier/text/express mail. For those patients completing a paper tool, a research staff member will contact the patient daily via phone call to obtain their daily assessment scores. Study team members will call patients at days 7, 14, 21, and day 28 of the study period to obtain information regarding hospitalizations, ER visits, additional medications prescribed by a healthcare provider

or over the counter, and any side effects from the study supplements that the patient could have experienced. Study team members may also call patients if non-compliance with completion of the daily symptom assessment tool.

Patients seen in the Emergency Department (ER) during the course of study participation will continue to take study supplement(s) (if within the initial 10 days) and continue daily symptom assessments as above.

Patients admitted to the hospital during the course of study participation will no longer be required to continue study supplementation nor track their daily symptoms.

2.1 Primary Objective

The primary objective will be evaluating the duration of symptoms in the four study arms. We will assess this by counting from when peak symptom score is achieved to when there is a 50% reduction in the symptom severity score.

3.0 Selection of Subjects

3.1 Inclusion/Exclusion Criteria

Inclusion:

A) 520 patients with a positive diagnosis of coronavirus 2019, managed in an outpatient setting, who are ≥18 years of age, divided into 4 groups as detailed above having any of the following symptoms

1. Fever or chills
2. Shortness of breath or difficulty breathing
3. Cough
4. Fatigue
5. Muscle or body Aches
6. Headache
7. New loss of taste

8. New loss of smell
9. Congestion or runny nose
10. Nausea
11. Vomiting
12. Diarrhea

B) Women of child bearing potential:

a. Have had a menstrual period within the past 30 days, or

b. Have had previous sterilization, or

c. Are perimenopausal (less than 1 year) who have a negative pregnancy test, or

d. Women of childbearing potential who do not meet the above and have a negative pregnancy test.

Exclusion:

A) Patients who are hospitalized when they are tested for COVID-2019

B) Patients who reside outside of Ohio or Florida

C) Pregnant women

   a. Current known pregnancy

   b. Positive pregnancy test (women of child bearing potential who have not had previous sterilization as defined as hysterectomy or tubal ligation. See above inclusion criteria)

D) Women of childbearing potential who do not meet the above criteria, last menstrual period greater than 30 days and have a positive pregnancy test.

E) Lactating women

F) End stage CKD (Chronic kidney disease)

G) Advanced liver disease awaiting transplant

H) History of Calcium Oxalate kidney stones.

4.0 Study Procedures

Prior to the initiation of participation, informed consent and permission to use protected health information will be obtained via virtual or phone consent as per Cleveland Clinic guidance, policies and procedures for COVID positive patients.

4.1 Study Evaluations

**Study Assessment**	**Screening/Baseline** (Visits may occur in any of the following platforms: Phone/Virtual/ Email) (Window -1 or +2 days)	**Daily through day 28** (Visits may occur in any of the following platforms: Phone/Virtual/ Email) (Window -1 or +2 days)	**Day 7** (Visits may occur in any of the following platforms: Phone/Virtual/ Email) (Window -1 or +2 days)	**Day 14** (Visits may occur in any of the following platforms: Phone/Virtual/ Email) (Window -1 or +2 days)	**Day 21** (Visits may occur in any of the following platforms: Phone/Virtual/ Email) (Window -1 or +2 days)	**Day 28** (Visits may occur in any of the following platforms: Phone/Virtual / Email) (Window -1 or +2 days)
**Informed Consent**	X					
**Medical History**	X					
**Symptoms**	X	X	X	X	X	X
**Medications Taking at Home**	X	X				
**Study Supplementation Tablet(s)**		X (May start on Day 0 or Day1)	X (Through day 10)			
**Emergency Room Visits**			X	X	X	X
**Hospitalizations**			X	X	X	X
**Change in Therapy**			X	X	X	X
**Side Effects**			X	X	X	
**Vital Status**						X

**At home urine pregnancy test (women of child bearing potential as per I/E criteria)**	X<br>*(Before taking first dose of study drug)					

5.0 Statistical Analysis and Endpoints

5.1 Sample Size:

This randomized control trial will aim to prospectively enroll 520 patients who have received a positive confirmed diagnosis of coronavirus 2019. All patients will be followed for 28 days to review vital status, hospitalizations, emergency room visits, and need for additional treatment. We assumed the standard of care group will have a 50% reduction of symptom severity in approximately 6± 3 days, and that at least one of the other 3 study groups will reach a 50% reduction in 5 ± 3 days. When the sample size in each of the 4 groups is 130, a one-way analysis of variance will have 80% power (2-sided alpha of 0.05) to detect a difference in means characterized by a variance of means, V, of 0.188, assuming that the common standard deviation is 3 days. Our study aims to enroll 520 patients to account for study drop out as well.

5.2 Primary Endpoints

The primary endpoint will be the number of days required to reach a 50% reduction in the symptom severity score from peak symptom score.

5.3 Secondary Endpoints

Additional endpoints to be considered include:

A) Number of days required to reach a total score of 0

B) Individual severity of illness scores (fever, cough, shortness of breath, fatigue)

C) Cumulative severity score at day 5 of the study

D) Emergency room visits

E) Hospitalizations

F) Adjunctive prescribed medications

G) Side effects of supplementation

5.4 Analytical Plan

Descriptive statistics will be generated for all baseline patient characteristics, complications, and endpoints. Frequency counts and percentages will be used to summarize binary and categorical data. Continuous data will be summarized using means ± standard deviation, median and interquartile range, and minimum/maximum values. ANOVA tests will be used to describe the difference in number of days to a 50% reduction on the symptom severity score between the four groups. All pairwise comparisons will be generated and adjusted for multiple comparisons using the Tukey method.

6.0 Safety Reporting

6.1 Definitions

6.1.1 Adverse Events

An adverse event (AE) is any event, side effect, or untoward medical occurrence in a subject in a clinical trial whether or not it is considered to have a causal relationship to the study drug, device or procedure. An AE can therefore be any unfavorable and unintended sign, symptom, laboratory finding outside of normal range, physical examination finding, or disease

temporally associated with the use of the study drug, device or procedure whether or not the event is considered related to the study drug, device or procedure.

6.1.2 Serious Adverse Events

Adverse events are classified as serious or non-serious. A ***serious adverse event*** is defined as any adverse event occurring that results in any of the following outcomes:

1) Death

2) A life-threatening experience

3) Inpatient hospitalization

4) A persistent or significant disability or incapacity

5) A congenital anomaly or birth defect

Important medical events that may not result in death, be life-threatening, or require hospitalization may be considered a serious adverse event when, based upon appropriate medical judgment, they may jeopardize the patient or subject and may require medical or surgical intervention to prevent one of the outcomes listed in this definition.

6.1.3 Documenting and Reporting of Adverse Events

For the purposes of this study adverse events that are in the opinion of the investigator, thought to be both serious, unexpected and related to study supplement(s) or procedures should be recorded in the database and reported to the IRB per current reporting policies and procedures. All patient deaths; regardless of causality or relatedness should be recorded in the database and reported to the IRB as per current IRB reporting policies.

We will have a study safety committee to evaluate data for an increased signal for adverse side-effects.

7.0 Administration

7.1 Maintaining the Security of Patient Data

Data will be stored on in a REDCAP database. There is an instance of this database maintained as a resource at Cleveland Clinic. REDCAP is maintained by Cleveland Clinic but is accessible on and off campus in secure fashion. We will analyze the data using SAS, version 9.4 or other programs as the discretion of the investigators or statisticians. The data will be entered into the database after completion of the procedure. Those members of the study team that have been delegated by the Principal Investigator to do so, are the only individuals that will have permission to enter and access the data in the study RedCap database. There is risk even with using secure database that personal health information that there may be loss of confidentiality. Furthermore, some disclosures of public health information will take place consistent with public health requirements as a part of the study.

7.2 Informed Consent

This is a minimal risk, study. The risks involve the potential risk from side effects from the study supplements. However since these are commercially available supplements without prescriptions and patients will only be taking for 10 days there should not be severe side effects. Reported side effects of ascorbic acid at higher doses of 6000-8000 mg are hyeroxaluria and formation of kidney stones in 1-10 percent of patients though occurs in patients taking high doses for long periods of time. Other side effects in less than 1 percent are diarrhea, flushing,

headache, heartburn, nausea/vomiting. Adverse effects of zinc in low risk include abdominal cramps, decrease appetite, diarrhea, epigastric pain, nausea/vomiting, tingling, numbness.

.**7.3 Investigator Assurances**

The Registry/Database PI is responsible for reviewing and authorizing all requests from others to obtain data from this database. Any additional users, known as Sub-Investigators, must submit to the PI a written request along with a signed Sub-Investigator Statement of Assurance. The PI is responsible for authorizing the release of data to Sub-Investigators and tracking these uses with a brief description of their activity.

The PI confirms no data from this database will be released without a written request from the sub-investigator and that the data provided will not contain identifiers unless consent from subjects has been obtained.

APPENDIX A: Symptom Assessment Tool

Day	1	2	3	4	5	6	7	8	9	10	11	12	13	14	15	16	17	18	19	20	21	22	23	24	25	26	27	28
(A) Temperature																												
(B) Shortness of breath																												
(C) Cough																												
(D) Fatigue																												
(E) Muscle/ body aches																												
(F) Headache																												
(G) New loss of taste																												
(H) New loss of smell																												
(I) Congestion/ runny nose																												
(J) Nausea																												
(K) Vomiting																												
(L) Diarrhea																												

KEY

A) Temperature Scale:	B) Cough Scale:	C) Shortness of breath:	D) Fatigue:	E) Muscle or body aches:	F) Headache:
0 = ≤98.6<br>1 = >98.6- 100.6<br>2 = > 100.6 – 102.6<br>3 = >102.6<br>Fever: (If no thermometer)<br>0= No fever & no chills,<br>1= No fever & chills	0 = no cough<br>1 = mild cough<br>2 = moderate cough<br>3 = severe cough	0 = no shortness of breath<br>1 = with moderate intensity exercise<br>2 = with walking on flat surface<br>3 = short of breath with	0 = No fatigue/ energetic<br>1=mild fatigue<br>2=moderate fatigue<br>3=severe fatigue	0 = No muscle or body aches<br>1=mild muscle or body aches<br>2=moderate muscle or body aches<br>3=severe muscle or	0 = No headache<br>1=mild headache<br>2=moderate headache<br>3=severe headache

2= Fever & no chills<br>3= Fever & chills		getting dressed or daily activities		body aches.	
**G) New loss of taste:**<br>0 = No loss of taste<br>1=mild loss of taste<br>2=moderate loss of taste<br>3=severe loss of taste	**H) New loss of smell:**<br>0 = No loss of smell<br>1=mild loss of smell<br>2=moderate loss of smell<br>3=severe loss of smell	**I) Congestion or runny nose:**<br>0 = No congestion or runny nose<br>1=mild congestion or runny nose<br>2=moderate congestion or runny nose<br>3=severe congestion or runny nose	**J) Nausea:**<br>0 = No nausea<br>1=mild nausea<br>2=moderate nausea<br>3=severe nausea	**K) Vomiting:**<br>0 = No vomiting<br>1=mild vomiting<br>2=moderate vomiting<br>3=severe vomiting	**L) Diarrhea:**<br>0 = No diarrhea<br>1=mild diarrhea<br>2=moderate diarrhea<br>3=severe diarrhea

8.0 References

1. Huang C, Wang Y, Li X, et al. Clinical features of patients infected with 2019 novel coronavirus in Wuhan, China. Lancet. 2020;395(10223):497-506.

2. Wu Z, Mcgoogan JM. Characteristics of and Important Lessons From the Coronavirus Disease 2019 (COVID-19) Outbreak in China: Summary of a Report of 72 314 Cases From the Chinese Center for Disease Control and Prevention. JAMA. 2020;

3. Hemila H, Chalker E. Vitamin C for preventing and treating the common cold (Review), Cochrane Database of Systematic Reviews 2017, (2).

4. Singh M, Das RR. Zinc for the Common Cold. Cochrane Database for Systematic Review 2011; (2).

5. Eby GA, Davis DR, Halcomb WW. Reduction in duration of common cold by zinc gluconate lozenges in a double-blind study. Antimicrobial Agents Chemotherapy.1984;25:20-24.

6. Harri Hermilla, Zinc lozenges and common cold: A meta- analysis comparison of zinc acetate and zinc gluconate and zinc dosage. JSRM Open, 2017; 8(5).

7. Carr AC. Vitamin C administration in the critically ill: a summary of recent meta-analyses. Crit Care. 2019;23(1):265.

8. Hemilä H. Vitamin C and Infections. Nutrients. 2017;9(4)

9. Hoffmann M, Kleine-weber H, Schroeder S, et al. SARS-CoV-2 Cell Entry Depends on ACE2 and TMPRSS2 and Is Blocked by a Clinically Proven Protease Inhibitor. Cell. 2020;

10. Maggini S, Beveridge S, Suter M. A combination of high-dose vitamin C plus zinc for the common cold. J Int Med Res. 2012;40(1):28-42.

11. Gammoh NZ, Rink L. Zinc in Infection and Inflammation. Nutrients. 2017;9(6).

12. Te velthuis AJ, Van den worm SH, Sims AC, Baric RS, Snijder EJ, Van hemert MJ. Zn(2+) inhibits coronavirus and arterivirus RNA polymerase activity in vitro and zinc ionophores block the replication of these viruses in cell culture. PLoS Pathog. 2010;6(11):e1001176.

13. Hemilä H. Vitamin C, respiratory infections and the immune system. Trends Immunol. 2003;24(11):579-80.

14. Atherton JG, Kratzing CC, Fisher A. The effect of ascorbic acid on infection chick-embryo ciliated tracheal organ cultures by coronavirus. Arch Virol. 1978;56(3):195-9.

15. Davelaar, F.G., Bos, J. (1992). Ascorbic Acid and infectious bronchitis infections in broilers. Avian Pathology 21,581-9.
