# Supplementary material for: Effect of High-Dose Zinc and Ascorbic Acid Supplementation vs Usual Care on Symptom Length and Reduction Among Ambulatory Patients With SARS-CoV-2 Infection: The COVID A to Z Randomized Clinical Trial
Source: JAMA Netw Open. 2021 Feb 12;4(2):e210369. doi: 10.1001/jamanetworkopen.2021.0369 (PMC7881357; doi:10.1001/jamanetworkopen.2021.0369)
Supplement: Supplement 2. — eTable. Adverse Effects in the Study Population [file jamanetwopen-e210369-s002.pdf]

Supplemental Online Content

Thomas S, Patel D, Bittel B, et al. Effect of high-dose zinc and ascorbic acid supplementation vs usual care on symptom length and reduction among ambulatory patients with SARS-CoV-2 infection: the COVID A to Z randomized clinical trial. *JAMA Netw Open*. 2021;4(2):e210369.
doi:10.1001/jamanetworkopen.2021.0369

eTable. Adverse Effects in the Study Population

This supplemental material has been provided by the authors to give readers additional information about their work.

© 2021 Thomas S et al. *JAMA Network Open.*

eTable. Adverse Effects in the Study Population

Parameter<br><br>n (%)	Total<br><br>(N=196)	Standard of Care (N=46)	Ascorbic acid Only (N=43)	Zinc Only (N=54)	Ascorbic acid + Zinc (N=53)	P-value
Experienced Side Effect from Study Assigned Supplements within Past 7 days	44 (22.4)	0 (0.0)	17 (39.5)	10 (18.5)	17 (32.1)	<0.001
None	103 (52.6)	3 (6.5)	25 (58.1)	42 (77.8)	33 (62.3)	<0.001
Flushing or redness of skin	0 (0.0)	0 (0.0)	0 (0.0)	0 (0.0)	0 (0.0)	N/A
Headache	5 (2.6)	0 (0.0)	1 (2.3)	0 (0.0)	4 (7.5)	0.028^{EX}
Nausea	15 (7.7)	0 (0.0)	6 (14.0)	4 (7.4)	5 (9.4)	0.052^{EX}
Vomiting	2 (1.0)	0 (0.0)	1 (2.3)	1(1.9)	0 (0.0)	0.593^{EX}
Tingling	2 (1.0)	0 (0.0)	1 (2.3)	1(1.9)	0 (0.0)	0.593^{EX}
Numbness	0 (0.0)	0 (0.0)	0 (0.0)	0 (0.0)	0 (0.0)	N/A
Stomach pains/cramps	11 (5.6)	0 (0.0)	5 (11.6)	1(1.9)	5 (9.4)	0.021^{EX}
Diarrhea	15 (7.7)	0 (0.0)	7 (16.3)	4 (7.4)	4 (7.5)	0.027^{EX}
Dizziness/faintness	2 (1.0)	0 (0.0)	1 (2.3)	0 (0.0)	1 (1.9)	0.471^{EX}
Other	7 (3.6)	0 (0.0)	1 (2.3)	1(1.9)	5 (9.4)	0.055^{EX}

^{NP}=non-paramatric test; ^{EX}=Exact test.

© 2021 Thomas S et al. *JAMA Network Open.*
