# Supplementary material for: Effect of High-Dose Zinc and Ascorbic Acid Supplementation vs Usual Care on Symptom Length and Reduction Among Ambulatory Patients With SARS-CoV-2 Infection: The COVID A to Z Randomized Clinical Trial
Source: JAMA Netw Open. 2021 Feb 12;4(2):e210369. doi: 10.1001/jamanetworkopen.2021.0369 (PMC7881357; doi:10.1001/jamanetworkopen.2021.0369)
Supplement: Supplement 3. — Data Sharing Statement [file jamanetwopen-e210369-s003.pdf]

Data Sharing Statement

Thomas. Effect of High-Dose Zinc and Ascorbic Acid Supplementation vs Usual Care on Symptom Length and Reduction Among Ambulatory Patients With SARS-CoV-2 Infection. *JAMA Netw Open*. Published February 12, 2021. doi:10.1001/jamanetworkopen.2021.0369

Data
Data available: No
